# Formation of NiFe_2_O_4_/Expanded Graphite Nanocomposites with Superior Lithium Storage Properties

**DOI:** 10.1007/s40820-017-0127-7

**Published:** 2017-02-21

**Authors:** Yinglin Xiao, Jiantao Zai, Bingbing Tian, Xuefeng Qian

**Affiliations:** 1 SZU-NUS Collaborative Innovation Center for Optoelectronic Science and Technology, Key Laboratory of Optoelectronic Devices and Systems of Ministry of Education and Guangdong Province, College of Optoelectronic Engineering, Shenzhen University, Shenzhen, 518060 People’s Republic of China; 2 School of Chemistry and Chemical Engineering, State Key Laboratory of Metal Matrix Composites, Shanghai Jiao Tong University, Shanghai, 200240 People’s Republic of China; 3 Department of Chemistry, National University of Singapore, 3 Science Drive 3, Singapore, 117543 Singapore

**Keywords:** NiFe_2_O_4_, Expanded graphite, Anode materials, Lithium-ion batteries

## Abstract

A NiFe_2_O_4_/expanded graphite (NiFe_2_O_4_/EG) nanocomposite was prepared via a simple and inexpensive synthesis method. Its lithium storage properties were studied with the goal of applying it as an anode in a lithium-ion battery. The obtained nanocomposite exhibited a good cycle performance, with a capacity of 601 mAh g^−1^ at a current of 1 A g^−1^ after 800 cycles. This good performance may be attributed to the enhanced electrical conductivity and layered structure of the EG. Its high mechanical strength could postpone the disintegration of the nanocomposite structure, efficiently accommodate volume changes in the NiFe_2_O_4_-based anodes, and alleviate aggregation of NiFe_2_O_4_ nanoparticles.

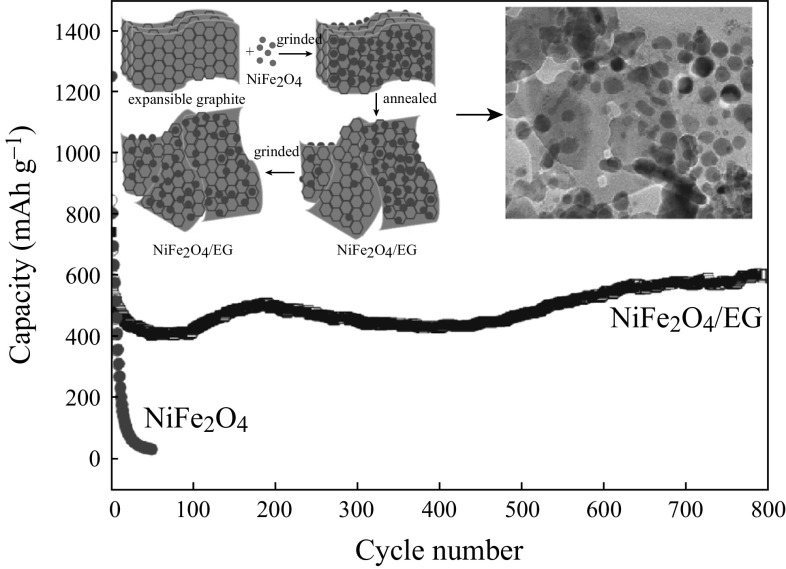

## Highlights

- A NiFe_2_O_4_/expanded graphite (NiFe_2_O_4_/EG) nanocomposite was synthesized via a simple grinding and mixing process followed by annealing at a high temperature. The obtained NiFe_2_O_4_/EG nanocomposite showed superior lithium storage properties, with a capacity of 601 mAh g^−1^ at a current density of 1 A g^−1^ after 800 cycles.
- The hybridNiFe_2_O_4_/EG nanostructure could efficiently improve the electrical conductivity and maintain structure stability, and its disintegration was delayed during discharge–charge processes, which led to a good cycling performance.

## Introduction

To meet the demands of high-energy storage for practical applications in electric vehicles (EVs) and hybrid electric vehicles (HEVs), much attention has been paid to lithium-ion batteries (LIBs) because of their distinct advantages, e.g., their large specific capacity, high energy density, long cycle life, and environmental friendliness [1–3]. Today, there is a great demand to design and develop novel and high-performance electrode materials to achieve LIBs with higher energy density, longer cycle life, improved safety, and lower cost. However, it is still urgency to improve the reversible charge capacity of anode materials in commercial LIBs, i.e., graphite [4, 5]. Among various anode materials with higher specific capacities than that of the material used in commercial batteries, ternary compounds (e.g., CuFe_2_O_4_ [6, 7], ZnFe_2_O_4_ [8, 9], CoFe_2_O_4_ [10–12], Zn_2_SnO_4_ [9, 13], ZnMn_2_O_4_ [14–17]) have been considered promising candidates because of their high theoretical capacities, low cost, and safety [9]. Nevertheless, their poor electrical conductivities and huge volume changes during continuous charge–discharge processes would lead to electrode pulverization. Rapid disintegration of these electrode materials caused by induced mechanical stress is responsible for a decrease in the capacity upon cycling and further hinders their practical applications [18, 19]. Expanded graphite (EG) possesses many advantageous properties, including fewer functional groups, better conductivity, higher mechanical strength [20], and lower cost [21]. A layered NiFe_2_O_4_/EG composite structure can be fabricated by incorporating nanostructured NiFe_2_O_4_ material with EG. This provides outstanding electron conductivity [22–25] and an ideal solution to the aforementioned inherent drawbacks of ternary compounds. However, to the best of our knowledge, there have been few reports on the construction of nanostructured material/EG composites with superior lithium storage properties because it is a challenge to homogenously disperse nanostructured materials into EG nanosheets.

In this work, a NiFe_2_O_4_/EG nanocomposite was easily fabricated via a grinding and mixing process, followed by annealing at a high temperature. This NiFe_2_O_4_/EG nanocomposite showed superior lithium storage properties, with a capacity of 601 mAh g^−1^ at a current density of 1 A g^−1^ after 800 cycles. In general, the combination of several structural features of the NiFe_2_O_4_/EG nanocomposite may contribute to the enhanced capacity and cycling performance. First, the layered structure of the NiFe_2_O_4_/EG nanocomposite can alleviate agglomeration of materials and improve the cycling performance. Second, the higher mechanical strength of EG can postpone disintegration of the nanocomposite structure. Third, EG can provide an enhanced conductivity performance, which is critical for the lithium storage performance. Fourth, EG has a high reversible capacity and good cycling performance, which can assist in enhancing the capacity and cycling performance of NiFe_2_O_4_/EG nanocomposite.

## Experimental

### Synthesis of NiFe_2_O_4_ and NiFe_2_O_4_/EG Nanocomposites

NiFe_2_O_4_ was synthesized using the procedure reported in a previous paper [26]. Typically, 1 mmol of nickel chloride hexahydrate (NiCl_2_·6H_2_O), 2 mmol of ferric chloride hexahydrate (FeCl_3_·6H_2_O), and 3 mL of ammonia were dissolved in 37 mL of alcohol. The mixture was sonicated for 30 min, transferred to a Teflon-lined autoclave, and then maintained at 180 °C for 12 h. The final product was separated by centrifugation and dried at 60 °C.

The NiFe_2_O_4_/EG nanocomposite was synthesized using the following procedure: 0.5 g of NiFe_2_O_4_, 0.2 g of expandable graphite, and 5 mL of alcohol were mixed and ground until the alcohol was completely volatilized. After that, the mixture was annealed at 1000 °C for 5 min in an Ar atmosphere, and the final product was ground and used as the active material for lithium-ion batteries.

### Characterizations

The crystal structures of the powder samples were characterized using X-ray diffraction (XRD, Shimadzu XRD-6000, CuKα, 40 kV, 30 mA, 20° ≤ 2θ ≤ 70°). A thermogravimetric (TG) analysis was performed on a PerkinElmer 7 instrument to determine the weight ratio of EG to NiFe_2_O_4_. The morphology of each sample was studied using a transmission electron microscopy (TEM) system (JEOL, JEM-2100).

### Electrochemical Testing

The working electrodes were fabricated from a slurry containing 80% active material, 10% polymer binder (polyvinylidene difluoride, PVDF), and 10% acetylene black on a copper foil using the procedure outlined in previous work [27] and dried in a vacuum oven at 80 °C for 12 h. The electrochemical performances were determined using a LAND battery tester (CT2001A model, Wuhan Jinnuo Electronics, Ltd.) between 0.01 and 3 V versus Li^+^/Li. All of the tests were performed at room temperature, with an electrolyte composed of 1 mol L^−1^ of LiPF_6_ in a mixed solvent of ethylene carbonate (EC)/diethylene carbonate (DEC) (1:1 vol%) and a Li cathode placed in the cell. Cyclic voltammetry measurements were performed using a CHI 660C potentiostat between 0.01 and 3 V at a scan speed of 0.5 mV s^−1^. A frequency range of 10 kHz to 0.1 Hz was used for electrochemical impedance spectroscopy (EIS) at an open-circuit potential with an alternating spectrum (AC) perturbation of 10 mV on a Zennium electrochemistry workstation.

## Results and Discussion

**Fig. 1:**
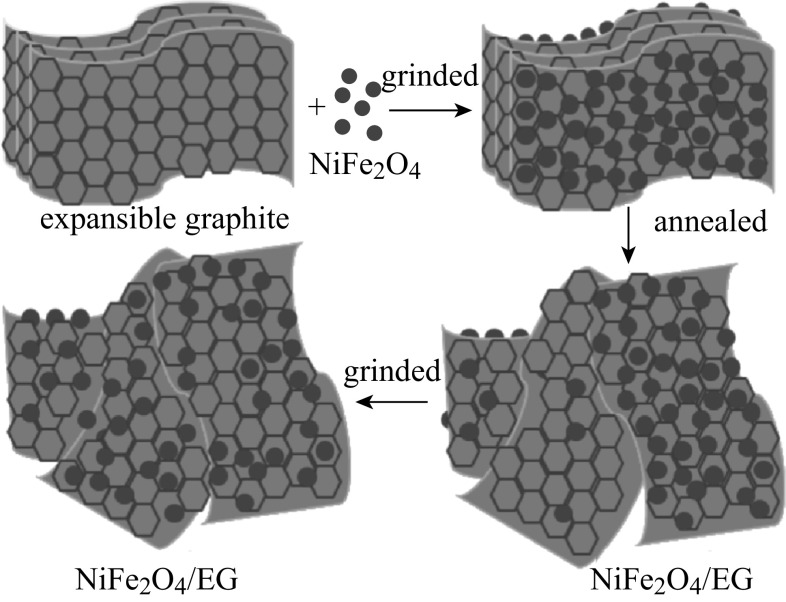
Schematic illustration of possible formation mechanism of NiFe_2_O_4_/EG nanocomposite

The synthesis procedure is schematically depicted in Fig. 1. In the first step, NiFe_2_O_4_ is homogenously dispersed onto the surface of the EG after the grinding process. After the calcination process, a mixture of thermal EG nanosheets and NiFe_2_O_4_ is obtained. Finally, NiFe_2_O_4_ is homogenously dispersed onto the surface of the EG nanosheets during the grinding process, producing the desired NiFe_2_O_4_/EG nanocomposites.

**Fig. 2:**
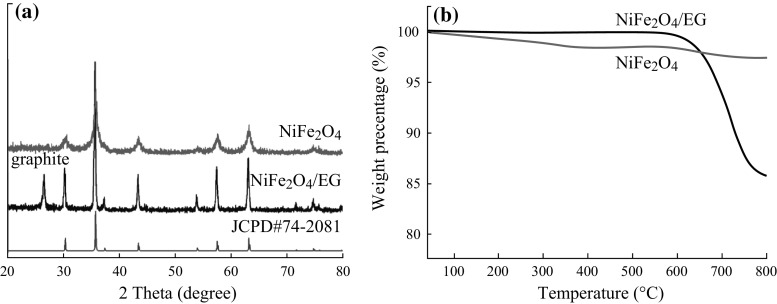
**a** XRD patterns and **b** TGA results for NiFe_2_O_4_ and NiFe_2_O_4_/EG

The XRD pattern of NiFe_2_O_4_ in Fig. 2a shows that all of the diffraction peaks can be readily indexed to cubic NiFe_2_O_4_ (JCPDS card no. 227, space group: Fd-3 m, *a* = 8.33790 Å). One additional diffraction peak located at approximately 26.2° in the XRD pattern of NiFe_2_O_4_/EG suggests the presence of EG. No other additional peaks are observed in the XRD patterns, suggesting high purity of the obtained products. The weight ratio of EG in the NiFe_2_O_4_/EG nanocomposite was evaluated using TGA in air (Fig. 2b). Assuming that the final residue was NiFe_2_O_4_, the lost weight of NiFe_2_O_4_/EG may correspond to the oxidation of EG to CO_2_. Based on the lost weight values of the NiFe_2_O_4_ and NiFe_2_O_4_/EG nanocomposites, the weight percentage of EG in the NiFe_2_O_4_/EG was approximately 14%.

**Fig. 3:**
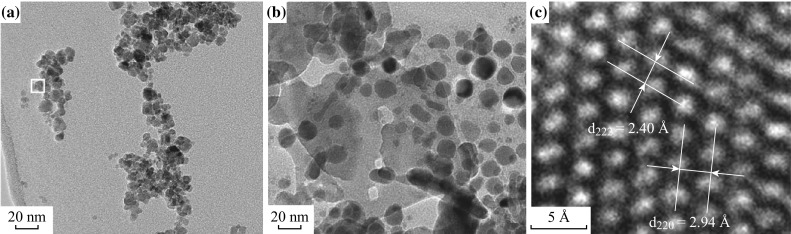
TEM images of **a** NiFe_2_O_4_ and **b** NiFe_2_O_4_/EG and HRTEM image of NiFe_2_O_4_

The morphologies and structures of the as-prepared NiFe_2_O_4_ and NiFe_2_O_4_/EG nanocomposites were studied using TEM. It was found that the size of the as-prepared NiFe_2_O_4_ was approximately 20 nm (Fig. 3a); NiFe_2_O_4_ was homogenously dispersed into the paper-like EG nanosheets in the NiFe_2_O_4_/EG nanocomposite (Fig. 3b). Furthermore, the structural characteristics of a typical NiFe_2_O_4_ nanoparticle with visible lattice fringes were observed in the HRTEM image (Fig. 3c). Inter-planar distances of 0.294 and 0.240 nm were measured, which were consistent with the (220) and (222) crystal planes of the cubic NiFe_2_O_4_ phase, respectively, confirming the crystalline structure of the obtained NiFe_2_O_4_.

**Fig. 4:**
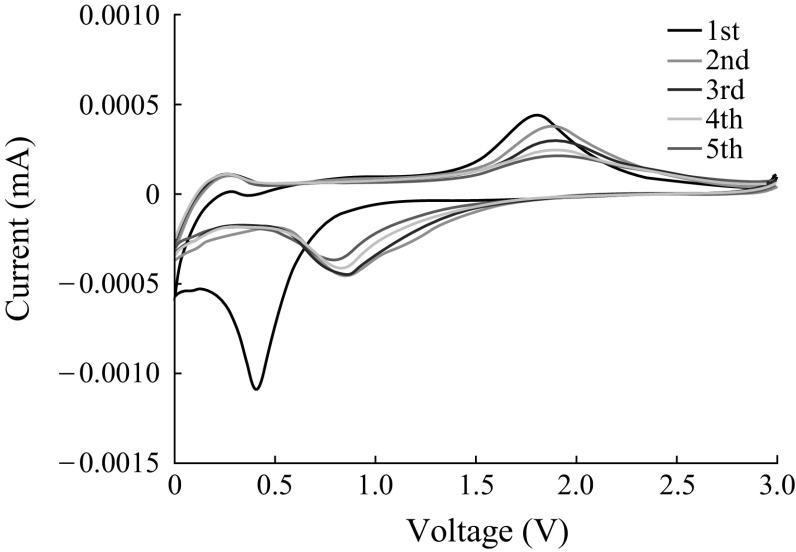
Cyclic voltammograms of NiFe_2_O_4_/EG nanocomposites for first five cycles between 3.00 and 0.01 V vs. Li, with scan rate of 0.5 mV s^−1^

The lithium storage properties of the NiFe_2_O_4_/EG nanocomposite as an anode material for LIBs were studied using CV between 0 and 3.0 V vs. Li/Li^+^ at a scan rate of 0.0005 V s^−1^ (Fig. 4). In the first CV cycle, the broad cathodic peak located at approximately 0.40 V could be attributed to the reduction reactions of Ni^2+^ to Ni^0^ and/or Fe^3+^ to Fe^0^. It is known that the first discharge plateaus of NiO and Fe_2_O_3_ are usually located at 0.26 and 0.55, respectively [28, 29]. Thus, the reduction peaks of NiFe_2_O_4_ could have overlapped to form a broad cathodic peak at 0.45 under our experimental conditions. This cathodic peak shifted to ~0.80 V in the second and subsequent cycles, corresponding to a lower polarization of the electrodes induced by the first lithiation–delithiation process. This process can be expressed by the following reaction:

1 $${\text{NiFe}}_{2} {\text{O}}_{4} + \, 8{\text{Li}}^{ + } + \, 8{\text{e}}^{ - } \to {\text{ Ni }} + \, 2{\text{Fe }} + \, 4{\text{Li}}_{2} {\text{O}}$$

The anodic peak located at 1.75 V in the first CV cycle may correspond to the oxidation of metallic Fe and Ni, and this anodic peak shifts to 1.90 V in subsequent cycles because of the polarization of the electrodes [3, 30]. This process can be expressed by the following reactions:

2 $${\text{Ni }} + {\text{ Li}}_{2} {\text{O }} \to {\text{ NiO }} + 2{\text{Li}}^{ + } + \, 2{\text{e}}^{ - }$$

3 $$2{\text{Fe }} + \, 3{\text{Li}}_{2} {\text{O }} \to {\text{ Fe}}_{2} {\text{O}}_{3} + \, 6{\text{Li}}^{ + } + 6{\text{e}}^{ - }$$

The reversible cathodic and anodic peaks located at approximately 0.80 and 1.90 V, respectively, in the second and subsequent cycles indicate a reversible oxidation–reduction reaction in the charge–discharge processes. To summarize, the reaction should be as follows:

4 $${\text{Ni }} + \, 2{\text{Fe }} + \, 4{\text{Li}}_{2} {\text{O }} \leftrightarrow {\text{ NiO }} + {\text{ Fe}}_{2} {\text{O}}_{3} + 8{\text{Li}}^{ + } + \, 8{\text{e}}^{ - }$$

Moreover, the observed small cathodic peaks at 0.1 V and anodic peaks located at 0.25 V may correspond to the reversible polymerization/oligomerization of carbonates and alkyl carbonates (the main components of the solid-state electrolyte interface), which would further lead to a reversible polymeric/gel film on the nanocomposite [31, 32].

**Fig. 5:**
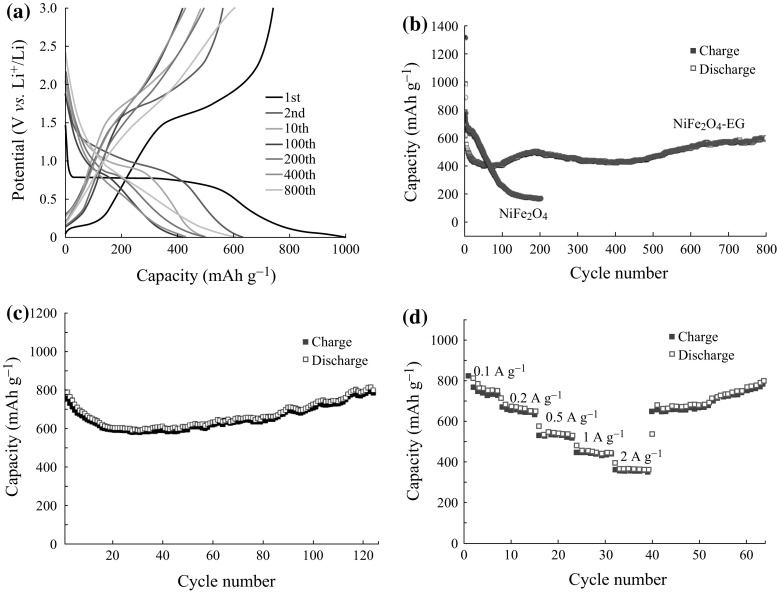
**a** Charge–discharge curves of NiFe_2_O_4_/EG at 0.1 A g^−1^, **b** cycling performances of NiFe_2_O_4_ and NiFe_2_O_4_/EG at current density of 1 A g^−1^, **c** cycling performance of NiFe_2_O_4_/EG at current density of 0.1 A g^−1^, and **d** rate capability of NiFe_2_O_4_/EG

The discharge–charge voltage profiles of the NiFe_2_O_4_/EG nanocomposite at a current density of 0.1 A g^−1^ are shown in Fig. 5a. The electrode based on NiFe_2_O_4_/EG had only one discharge platform, located at 0.75 V, which is attributed to the reduction reaction of NiFe_2_O_4_ with lithium during the first discharge process and is also associated with the formation of a solid-state electrolyte interface (SEI) [4]. The plateau increased to ~1.0 V in the subsequent cycles, in agreement with the galvanostatic discharge–charge data [33]. After the 10th cycle, the plateau at approximately 1.0 V had evolved into a slope, which may have been caused by Li^+^ trapping in the electrode during cycling [34]. The fact that the specific capacity was greater than 0.75 V in the first cycle and greater than 1.0 V in the subsequent cycles could be ascribed to the faradic capacitance on the surface or edge site of the EG. A similar phenomenon can be found in graphene nanosheets [35]. The fact that the capacity was less than 0.75 V in the first cycle and less than 1.0 V in subsequent cycles was mainly associated with the conversion–deconversion process of the binary oxide and the formation of the SEI layer [36]. The excellent mechanical property of EG can postpone the disintegration of the nanocomposite structure during the discharge–charge processes and leads to a good cycle performance [37, 38].

Figure 5b shows the cyclic performances of NiFe_2_O_4_ and NiFe_2_O_4_/EG at a current density of 1 A g^−1^. A discharge capacity of 986 mAh g^−1^ and a charge capacity of 741 mAh g^−1^ in the first cycle were observed on the NiFe_2_O_4_/EG electrode. The large initial irreversible discharge capacity could be attributed to the formation of the SEI. The capacities continued to decrease until the 50th cycle. This capacity decrease was partially ascribed to the decomposition of the electrolyte [39], along with the incompletely reversible reaction of the NiFe_2_O_4_/EG nanocomposite. It is interesting to note that after the 100th cycle, the reversible capacities significantly increased with the further activation of NiFe_2_O_4_, which was also observed in earlier work [40]. Furthermore, the reversible capacities of the NiFe_2_O_4_/EG continued to increase from 443 mAh g^−1^ in the 450th cycle to 601 mAh g^−1^ in the 800th cycle. These increasing capacities can be attributed to the reversible polymeric/gel film on the nanocomposite. A similar phenomenon has been observed in other transition metal oxides [37, 41]. The cyclic stability of the NiFe_2_O_4_/EG nanocomposite electrode was better than those reported in previous papers [42, 43]. The results indicated that the excellent mechanical properties of the EG contributed to the cyclic stability of the obtained NiFe_2_O_4_/EG composite.

**Fig. 6:**
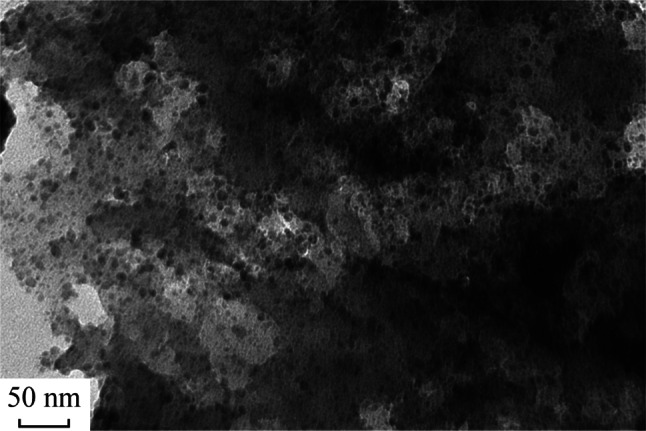
TEM images of NiFe_2_O_4_/EG nanocomposite after 120 discharge–charge processes

The cycling performance of the NiFe_2_O_4_/EG at a current density of 0.1 A g^−1^ up to 120 cycles is shown in Fig. 5c. The initial discharge capacity (1182 mAh g^−1^) was much higher than the charge capacity (790 mAh g^−1^), with a high irreversible capacity (a Coulombic efficiency of 66.8%) related to the formation of an SEI layer and a non-fully reversible conversion–deconversion process in the first lithiation–delithiation cycle. In subsequent cycles, the discharge and charge capacities were almost equal, with a Coulombic efficiency of ∼100%, which indicated excellent electrochemical reversibility. The capacity decreased slightly from the second to 20th cycle because of the non-fully reversible conversion–deconversion process and then slowly increased from the 21st to 120th cycle because of the reactivation of the NiFe_2_O_4_. The NiFe_2_O_4_/EG electrode material was collected after the 120th discharge–charge process and further analyzed using TEM (Fig. 6). The TEM image showed that the EG in the NiFe_2_O_4_-EG still had a layered structure and there was no agglomeration of NiFe_2_O_4_ nanoparticles, which was responsible for the good stability of the electrode. To further evaluate the stability of the NiFe_2_O_4_/EG nanocomposite, the rate capability was investigated at different rates from 0.1 to 2 A g^−1^ (Fig. 5d). The electrode had charge capacities of 824, 667, 529, and 445 mAh g^−1^ at current densities of 0.1, 0.2, 0.5, and 1.0 A g^−1^, respectively. Furthermore, at a current density of 2.0 A g^−1^, the NiFe_2_O_4_/EG electrode had a stable charge capacity of 361 mAh g^−1^, indicating that the obtained NiFe_2_O_4_/EG nanocomposite exhibited a remarkable high lithium storage capacity at a high rate.

**Fig. 7:**
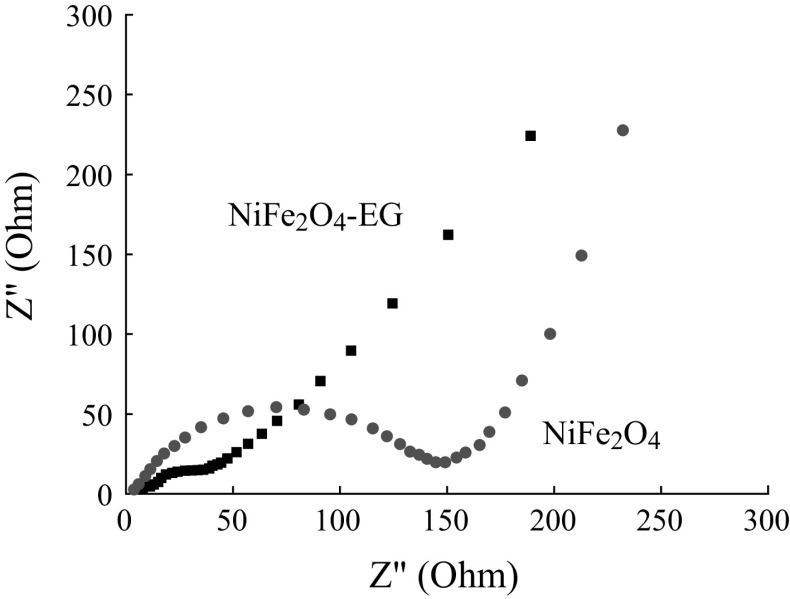
Nyquist plots of NiFe_2_O_4_ and NiFe_2_O_4_/EG at 0.08 V vs. Li after five cycles

To further investigate the effects of the EG in the NiFe_2_O_4_/EG nanocomposite, the conductivities of NiFe_2_O_4_ and NiFe_2_O_4_/EG after five discharge–charge cycles were evaluated using EIS measurements (Fig. 7). As widely discussed in EIS studies of intercalation–deintercalation-type materials, the semicircle in the medium-frequency region is assigned to the charge-transfer impedance, which is related to the electrochemical reaction between the electrolyte and electrode [6, 44]. In the plots for NiFe_2_O_4_ and its nanocomposite, the semicircle for the NiFe_2_O_4_/EG nanocomposite is smaller than that of NiFe_2_O_4_, indicating that the EG could facilitate the electron transfer between the electrolyte and NiFe_2_O_4_ and further improve the rate capability [45].

Based on the above discussion, the advantage of the NiFe_2_O_4_/EG nanocomposite involves its unique structural and electrochemical nature. First, the layered structure of the NiFe_2_O_4_/EG nanocomposite can alleviate the agglomeration of materials and improve the cycling performance. Second, EG has fewer functional groups and excellent mechanical properties, which can maintain the structure stability and postpone the disintegration of the nanocomposite structure during the discharge–charge processes, leading to a good cycle performance. Third, the high electrical conductivity of EG can increase the conductivity of the electrodes, ensuring fast electron transportation. Finally, EG has a high reversible capacity and good cycle performance, which is conducive to enhancing the capacity and cycling performance [46].

## Conclusions

We developed a simple method for the direct homogeneous dispersion of NiFe_2_O_4_ nanoparticles onto EG nanosheets for use as a superior anode material for lithium-ion batteries. This hybrid nanostructure showed improved electrical conductivity, maintained structure stability, and exhibited delayed disintegration during the discharge–charge processes, which led to a good cycle performance. As a result, the fabricated NiFe_2_O_4_/EG composite demonstrated a high reversible capacity of 601 mAh g^−1^ over 800 cycles at a current density of 1 A g^−1^. Our synthesis approach could easily be extended to combine other ternary compounds (MFe_2_O_4_, MCo_2_O_4_, MMn_2_O_4_, etc.) with EG, offering promising routes for the low-cost mass production of advanced electrode materials for the next generation of LIBs.
